# Complex contaminant mixtures and their associations with intima-media thickness

**DOI:** 10.1186/s12872-019-1246-5

**Published:** 2019-12-12

**Authors:** Eric N. Liberda, Aleksandra M. Zuk, Leonard J. S. Tsuji

**Affiliations:** 1 School of Occupational and Public Health, Ryerson University, Toronto, 350 Victoria St, Toronto, Ontario M5B 2K3 Canada; 2 Health Studies, and the Department of Physical and Environmental Sciences, University of Toronto Scarborough, Toronto, M1C 1A4 Ontario Canada

**Keywords:** Carotid intima media thickness, IMT, Contaminants, Exposure, Indigenous

## Abstract

**Background:**

The burden of cardiovascular disease (CVD) morbidity and mortality is higher among Indigenous persons, who also experience greater health disparities when compared to non-Indigenous Canadians, particularly in remote regions of Canada. Assessment of carotid intima-media thickness (cIMT), a noninvasive screening tool and can be used as biomarker to assess increased CVD risk. Few studies have examined environmental contaminant body burden and its association with cIMT.

**Methods:**

Data from the Environment-and-Health Study in the *Eeyou Istchee* territory of northern Québec, Canada was used to assess complex body burden mixtures of POPs, metals and metalloids among (*n* = 535) Indigenous people between 15 and 87 years of age with cIMT. First, Principal Component Analysis (PCA) was used to reduce the complexity of the contaminant data. Second, based on the underlying PCA profiles from the biological data, we examined each of the prominent principal component (PC) axes on cIMT using multivariable linear regression models. Lastly, based on these PC axes, cIMT was also regressed on summed (Σ) organic compound concentrations, polychlorinated biphenyl, perfluorinated compounds, respectively, ∑10 OCs, ∑13 PCBs, ∑3PFCs, and nickel.

**Results:**

Most organochlorines and PFCs loaded primarily on PC-1 (53% variation). Nickel, selenium, and cadmium were found to load on PC-5. Carotid-IMT was significantly associated with PC-1 *β* = 0.004 (95 % *CI* 0.001, 0.007), and PC-5 *β* = 0.013 (95 % *CI* 0.002, 0.023). However, the association appears to be greater for PC-5, accounting for 3% of the variation, and mostly represented by nickel. Results show that that both nickel, and ∑3PFCs were similarly associated with cIMT *β* = 0.001 (95 % *CI* 0.0003, 0.003), and *β* = 0.001 (95 % *CI* 0.0004, 0.002), respectively. But ∑10OCs was significantly associated with a slightly greater *β* = 0.004 (95 % *CI* 0.001, 0.007) cIMT change, though with less precision. Lastly, ∑13PCBs also increased *β* = 0.002 (95 % *CI* 0.0004, 0.003) cIMT after fully adjusting for covariates.

**Conclusion:**

Our results suggest that environmental contaminants are associated with cIMT. This is important for the Cree from the *Eeyou Istchee* territory who may experience higher body burdens of contaminants than non-Indigenous Canadians.

## Background

Cardiovascular disease (CVD) mortality has decreased globally in many regions [1]. However, the burden of CVD morbidity and mortality is higher among Indigenous persons, who also experience greater health disparities when compared to non-Indigenous Canadians [2–4]. While a variety of social, economic, and cultural factors may help explain why a decrease in CVD has not been observed among Indigenous communities [5–7], very little research has explored environmental contaminant exposures and the contribution to cardiovascular outcomes.

Indigenous community residents from coastal and inland Cree communities from the eastern James Bay region of subarctic Quebec, Canada, experience higher body burdens of environmental contaminants than non-Indigenous Canadians [8, 9]. In remote Indigenous communities, residents experience difficulties surrounding medical access, which may further compound health inequities [6] leading to increased risk of CVD morbidity and mortality. The use of carotid intima-media thickness (cIMT) measurements in remote locations can aid in earlier identification of individuals with emerging cardiovascular diseases without the need to travel out of the community to access a medical facility.

Carotid IMT is shown to be significantly correlated with a variety of cardiovascular measures and risk factors [10–13]. Therefore, assessing cIMT and its application in remote regions may particularly be useful as a non-invasive screening tool. In addition to the associations between cIMT and cardiovascular risk, various studies have examined the role of xenobiotics (e.g., air pollutant components) and CVD (e.g., [14–18]). Additionally, some studies have examined environmental contaminant body burden and its association with cIMT. Carotid IMT has been shown to be positively associated with various individual contaminants, including metals [19–22], persistent organic pollutants (POPs) [23], perfluorinated compounds (PFCs) [24–26], and bisphenol-A and phthalates [28]. However, no studies have assessed the effects of complex xenobiotic mixtures such as persistent organic pollutants (POPs), metals, and metalloids [28]. Therefore, the aim of this study was to assess the association between cIMT and complex body burden mixtures of persistent organic pollutants, metals, and metalloids using data from the Environment-and-Health Study in the *Eeyou Istchee* territory of Quebec, Canada.

## Methods

### Data sources

**Fig. 1:**
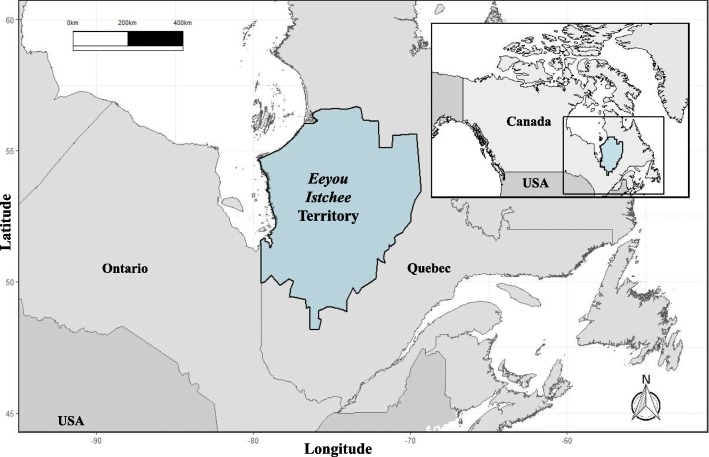
Location of the Eeyou Istchee Territory in Quebec, Canada. (Image generated using R [41] with GADM and mapdata packages [9, 16])

The *Eeyou Istchee* traditional territory consists of First Nations communities from the Eastern James Bay region, Quebec, Canada who are represented by the Grand Council of The Crees. These community members live in varying degrees of isolation, with the furthermost only being accessible by airplane or boat (Fig. 1). As part of the *Nituuchischaayihtitaau Aschii* – Multi-Community Environment-and-Health Study in *Eeyou Istchee*, participants answered personal and clinical questionnaires related to lifestyle, occupation, socio-demographic status, as well as dietary habits. The questionnaire was developed for this study and details were previously published by Nieboer et al. [32]. In total, there were 1730 participants representing all 9 communities who completed all, or portions of, the questionnaires and provided blood samples. Written informed consent was obtained from all participants or their guardians in Cree, English, or French. All work was approved by the research ethics boards of McGill University and Laval University, in partnership with the Cree Board of Health and Social Services of James Bay and McMaster University.

### Study population

Using the initial 1730 recruited participants from all 9 Indigenous communities, we excluded persons younger than 14 years of age and those with any CVD diagnoses from the analysis. Adolescents were included in the analysis because cIMT has been validated at younger ages [33]. Participants with a complete set of observations were retained and the final analytical sample included 535 participants (299 females and 236 males). As this study took place in remote locations, we collected data from each community beginning in 2002 and ended in 2009. Of the nine communities, two were sampled between 2002 to 2005, one in 2005, two in 2007, two in 2008, and the remaining two in 2009. At the request of the participants and the Cree Board of Health and Social Services James Bay we have not presented disaggregated information regarding the individual communities.

### Main outcomes

#### Ultrasound evaluation of the carotid artery

cIMT measurements of the carotid arteries were performed by two experienced sonographers on a high-resolution B-mode ultrasound machine (LogiqBook, GE Medical Systems, Milwaukee, WI) equipped with a linear 4–10 MHz probe (10LB-Rs). Plaque was identified and excluded from analysis by a transverse scan prior to cIMT scanning. A dozen 1-cm segments were scanned at the near and far walls of the carotid arteries and assessed using a dedicated image-analysis system (Carotid Analyzer for Research version 5.5.6, Medical Imaging Application, Coralville, IA). The average of the segmental means of the near and far walls of both left and right common carotid arteries was reported as the cIMT value for each individual as recommended by the American Society of Echocardiography [34].

### Exposure variables

#### Organics analysis

The analytical method for the organic pollutant analysis has been fully reported by Liberda et al. [8]. Briefly, the concentration of 15 polychlorinated biphenyl congeners (PCB; 28, 52, 99, 101, 105, 118, 128, 138, 153, 156, 163, 170, 180, 183, and 187), 13 chlorinated pesticides and their metabolites (Aldrin, ß-Hexachlorocyclohexane [HCH], α-Chlordane, γ-Chlordane, oxy-Chlordane, trans-Nonachlor, cis-Nonachlor, diphenyltrichloroethane [p,p′-DDT], dichlorodiphenyldichloroethylene [p,p′-DDE], Hexachlorobenzene [HCB], Mirex, Toxaphene 26, and Toxaphene 50), and 5 brominated organic compounds (polybrominated biphenyl-153; PBB 153, and four polybrominated diphenyl ethers; PBDE 47, PBDE 99, PBDE 100, PBDE 153) were determined. All samples were assessed on a gas chromatography–mass spectrometer (GC-MS) at the Institut National de Santé Publique du Québec (INSPQ), the reference laboratory for the Arctic Monitoring and Assessment Program (Agilent 6890 gas chromatograph equipped with an Agilent G2397A ECD and an Agilent 5973 network mass detector). Three PFCs, perfluorooctanoic acid (PFOA), perfluorooctane sulfonic acid (PFOS), and perfluorohexane sulfonic acid (PFHxS) were also measured at the INSPQ by using an alkaline extraction method with methyl-tert butyl ether and tetrabutylammonium hydrogen-sulfate before being assessed using Ultra Performance Liquid Chromatography (UPLC Waters Acquity) with a tandem mass spectrometer (MS/MS Waters Xevo TQ-S) (Waters; Milford, MA, USA) in the Multiple Reaction Monitoring mode with an electrospray ion source in the negative mode. The detection limits for the PFCs were 0.1 μg/L.

#### Metals and metalloid analysis

As with the organic contaminants, we have previously reported the methods, limits of detections, and other QA/QC data related to metals and metalloids [9, 35]. Briefly, whole blood samples were thawed and assessed for lead, cadmium, mercury, selenium, cobalt, copper, molybdenum, nickel, and zinc. Inorganic arsenic was assessed in urine and all contaminants were measured using a Perkin Elmer Sciex Elan 6000 inductively coupled plasma mass spectrometer (ICP-MS).

### Risk factor assessment

Demographic information (i.e., age, sex), and behavioral risk factors such as smoking habits were obtained via self-report through interviewer administered health questionnaires. Fasting blood samples were drawn to measure cardiometabolic variables during a physical examination by trained registered nurses. Anthropometric measures, included standing height and weight for body mass index (BMI) values. Blood pressure measurements (measured in millimeters of mercury, mm Hg) were examined according to a standardized protocol, where three separate measures were taken and the mean blood pressure value was calculated using the two last measurements. All participants rested for 5 min prior to measurements and had not smoked for at least 30 min.

#### Inflammatory and blood lipid marker measurements

Whole blood was collected for laboratory analysis of inflammatory and lipid markers. Tumor necrosis factor (TNF-α), high sensitivity c-reactive protein (hs-CRP), oxidized low-density lipoprotein (ox-LDL), Low-density lipoproteins (LDL), apolipoprotein-B (apo-B), and triglycerides were selected based on their biological roles in CVD.

TNF-α (R&D Systems, Minneapolis, MN), hs-CRP (BN-100 nephelmometer, Dade Behring, Deerfield, IN), and ox-LDL (Mercodia AB, Uppsala, Sweden) were assessed in blood plasma by ELISA following their respective manufacturers’ directions. Triglycerides, total cholesterol, and high-density lipoproteins (HDL) were measured on the Vitro 950 Chemistry Station (Ortho-Clinical Diagnostics, Raritan, NJ) as per the manufacturer’s directions. LDL was calculated based on the subtraction of HDL from the total cholesterol measurements. Apo-B was assessed on a protein analyzer as per the manufacturer (BN ProSpec System, Dade Behring).

### Statistical methods

#### Principal component analysis

Due to the high number of contaminants under assessment (43 in total), we performed a scaled and centered principal component analysis (PCA) to reduce the variables into a smaller number of uncorrelated predictor variables. Individual participant PC scores were generated from their contaminant loadings and used to create a PCA of the 43 contaminants which was then centered and scaled. Prior to principal component generation, the contaminant concentrations were transformed as log10 (1 + [μg/kg]) to improve the normality of the data distribution and remove negative values [36, 37]. PCs were selected based on eigenvalues greater than one. Non-detections in the contaminant data were input as half of the detection limit. A description on the use and merits of PCAs in environment and health projects can be found in Liberda et al. [8] and Wainman et al. [38].

#### Statistical analysis

Descriptive statistics of variables are reported, and where appropriate, means ± standard deviations (SD) are presented, otherwise frequencies and percentages are shown for categorical variables. Geometric means are also presented for skewed distributions.

Multivariable linear regression modeled the outcome, carotid IMT, on PCA environmental contaminants, adjusting for the a priori covariates. Three models are presented that explain the observed variation in cIMT by PCA contaminants. Model 1 represents a minimally adjusted linear regression of 5 PC axes on cIMT with age and sex as covariates. Model 2 represents the moderately adjusted regression with the same covariates as Model 1, but also including smoking status, BMI, and systolic blood pressure. Model 3 represents the fully adjusted model, which includes the following covariates: age, sex, smoking status, BMI, systolic blood pressure, LDL, Apo-B, triglycerides, TNF-α, hs-CRP, and ox-LDL. All covariates were assessed for multicollinearity, and residual plots were used to validate assumptions of linearity, normality and homoscedasticity.

Based on the multivariable linear regression findings between PCA contaminants and cIMT, we further performed analysis on nickel and the sum (∑) of the following compounds: organochlorines (∑10 OCs), polychlorinated biphenyls (∑13 PCBs), and perfluorinated compounds (∑3PFCs). These contaminants were selected based on significant associations between PC axes loadings and cIMT.

The significant associations between PC axes loadings and cIMT were observed and we performed further analysis on the sum (∑) of following organic compound concentrations (∑10 OCs), polychlorinated biphenyl compounds (∑13 PCBs), perfluorinated compounds (∑3PFCs) and nickel. Lastly, as additional sensitivity analysis, we restricted to adults > 30 years of age to examine the contribution of complex body burden mixtures on cIMT and found no differences between the group (data not shown).

All significance values were adjusted for multiple comparisons using the Holm method [39]. Associations were considered significant when the adjusted *p* < 0.05. All statistical analyses were conducted using R (version 3.5.0; Vienna, Austria).

## Results

### Descriptive results

Summary statistics of all risk factors and contaminants are presented in Table 1.

**Table 1:** Baseline descriptive parameters of participant cardiometabolic risk factors and contaminant body burdens from the *Nituuchischaayihtitaau Aschii* – Multi-Community Environment-and-Health Study

Characteristics	Population total (N)				
	535				
Demographic data					
Sex (% males)	236 (44%)				
Health behaviors					
Smoking status current smoker	278 (52%)				
Cardiometabolic Risk Factors	Mean	Geometric Mean	SD	Minimum value	Maximum value
Age (years)	38.5	35.3	15.7	15.0	87.0
IMT (mm)	0.7	0.6	0.2	0.4	2.0
BMI (kg/m^2^)	32.6	32.0	6.5	16.9	58.9
Apo B (g/L)	0.9	0.8	0.3	0.3	2.3
Triglycerides (mmol/L)	1.5	1.3	0.8	0.4	6.0
LDL (mmol/L)	2.7	2.6	0.8	0.9	5.5
Systolic BP (mm Hg)	120.7	119.8	15.1	80.0	198.0
hs-CRP (mg/L)	4.9	2.7	6.3	0.1	86.9
TNF- *α* (pg/mL)	3.6	2.8	3.6	0.5	42.0
Ox-LDL (U/I)	50.3	48.2	14.9	19.3	129.1
Contaminants					
Arsenic (Inorganic; μmol/L)	0.07	0.06	0.08	<DL	0.98
β-HCH (μg/L)	0.02	0.01	0.02	<DL	0.13
Cadmium (nmol/L)	14.18	8.06	14.22	<DL	73.00
cis-Nonachlor (μg/L)	0.04	0.01	0.07	<DL	0.56
Cobalt (nmol/L)	4.28	3.38	10.05	0.465	230.00
Copper (μmol/L)	15.27	15.10	2.42	10	27.00
HCB (μg/L)	0.10	0.06	0.14	<DL	1.30
Lead (μmol/L)	0.23	0.15	0.25	0.016	2.50
Mercury (nmol/L)	34.15	12.42	62.25	<DL	650.00
Mirex (μg/L)	0.26	0.05	0.51	<DL	4.10
Molybdenum (nmol/L)	6.15	5.56	3.12	<DL	26.00
Nickel (nmol/L)	21.84	19.56	10.03	<DL	110.00
Oxychlordane (μg/L)	0.07	0.03	0.11	<DL	0.97
PBB-153 (μg/L)	0.02	0.01	0.03	<DL	0.25
PBDE-100 (μg/L)	0.02	0.01	0.02	<DL	0.13
PBDE-153 (μg/L)	0.04	0.02	0.04	<DL	0.33
PBDE-47 (μg/L)	0.06	0.04	0.08	<DL	0.96
PBDE-99 (μg/L)	0.02	0.01	0.02	<DL	0.20
PCB-101 (μg/L)	0.02	0.02	0.00	<DL	0.04
PCB-105 (μg/L)	0.04	0.01	0.09	<DL	1.10
PCB-118 (μg/L)	0.23	0.06	0.53	<DL	7.30
PCB-128 (μg/L)	0.01	0.01	0.01	<DL	0.08
PCB-138 (μg/L)	0.57	0.16	1.02	<DL	9.60
PCB-153 (μg/L)	1.35	0.36	2.40	<DL	20.00
PCB-156 (μg/L)	0.16	0.04	0.30	<DL	2.40
PCB-163 (μg/L)	0.23	0.06	0.44	<DL	3.80
PCB-170 (μg/L)	0.32	0.08	0.56	<DL	4.10
PCB-180 (μg/L)	1.16	0.28	2.09	<DL	15.00
PCB-183 (μg/L)	0.11	0.03	0.18	<DL	1.60
PCB-187 (μg/L)	0.43	0.10	0.77	<DL	5.90
PCB-99 (μg/L)	0.10	0.04	0.19	<DL	1.90
PFHDxS (μg/L)	2.44	1.51	2.81	0.1	26.00
PFOA (μg/L)	3.31	2.55	2.53	<DL	17.00
PFOS (μg/L)	12.19	7.53	14.09	0.15	110.00
p’p’-DDE (μg/L)	2.39	1.09	3.71	0.045	39.00
p’p’-DDT (μg/L)	0.03	0.03	0.02	0.025	0.14
Selenium (μmol/L)	2.19	2.17	0.32	<DL	4.90
Toxaphene-26 (μg/L)	0.01	0.01	0.02	<DL	0.15
Toxaphene-50 (μg/L)	0.01	0.01	0.02	<DL	0.15
trans-Nonachlor (μg/L)	0.14	0.05	0.23	<DL	2.10
Zinc (μmol/L)	96.99	96.22	12.24	63	150.00

Standard deviation (SD)

The mean age of participants was 38.5 (± 15.7 years), and just over half (55%) were female. The average cIMT measurement was 0.673 mm (± 0.193 mm). As is common with environmental contaminants, the range of concentrations varied greatly.

### Contaminant PCA loadings

**Fig. 2:**
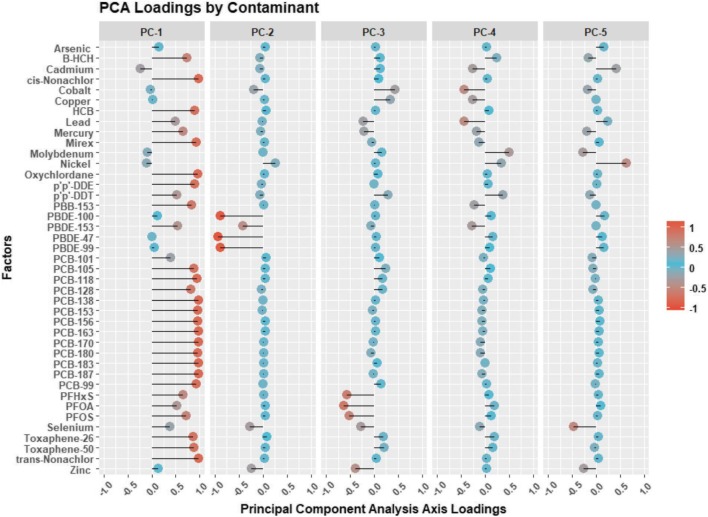
PCA loadings

The contaminant PC (principal component) loadings scores, which were generated from the individual PC scores are presented in Fig. 2. PC-1 (52.93% of the original variation explained) resulted in relatively high loadings for most organochlorines and PCBs. PC-2 (7.10% of the variation explained) was highly (negatively) loaded for the PBDEs. PC-3 (5.07% of the variation explained) was loaded for the PFCs (negatively), and PC-4 (3.71% of the variation explained) and PC-5 (3.10% of the variation explained) were loaded for various metals including nickel, selenium, and cadmium.

### cIMT and PCA

Results from the multivariable linear regression are shown in Table 2. Carotid IMT was significantly associated with PC-1 and PC-5 in all three models. PC-1, which represents mostly organochlorine contaminants increased cIMT by *β* = 0.004 (95 % *CI* 0.001, 0.007). However, the association appears to be greater for PC-5 *β* = 0.013 (95 % *CI* 0.002, 0.023), which accounts for 3% of the variation, and is mostly represented by nickel.

**Table 2:**
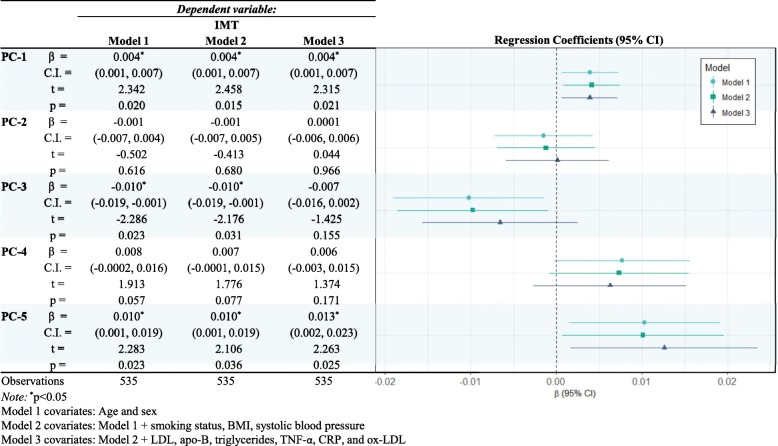
Multivariable linear regression models for the association between Carotid IMT and PCA of contaminant among participants from the *Nituuchischaayihtitaau Aschii* – Multi-Community Environment-and-Health Study

Carotid IMT was significantly and negatively associated with PC-3 (i.e., represented primarily by the PFCs) in the model 1 and model 2, *β* =  − 0.010 (95 % *CI* − 0.019, −0.001), but after fully adjusting for confounders the association was no longer significant. Important for interpretation, PFCs are negatively loaded within PC-3 and negatively associated with cIMT and therefore, should be interpreted positively. No models were significant for PC-2 or PC-4 with cIMT. PFCs are also moderately represented in PC-1.

### cIMT and contaminants

Sum of organic compound concentrations are presented in Table 3. Both nickel, and ∑3 PFCs were significantly and consistently associated with cIMT, (*β* = 0.001) across all three linear regression models. The sum of ∑10 OCs was significantly associated with a slightly greater *β* = 0.004 (95 % *CI* 0.001, 0.007) change in cIMT, though with less precision. Lastly, ∑13 PCBs also increased *β* = 0.002 (95 % *CI* 0.0004, 0.003) cIMT after fully adjusted for covariates.

**Table 3:**
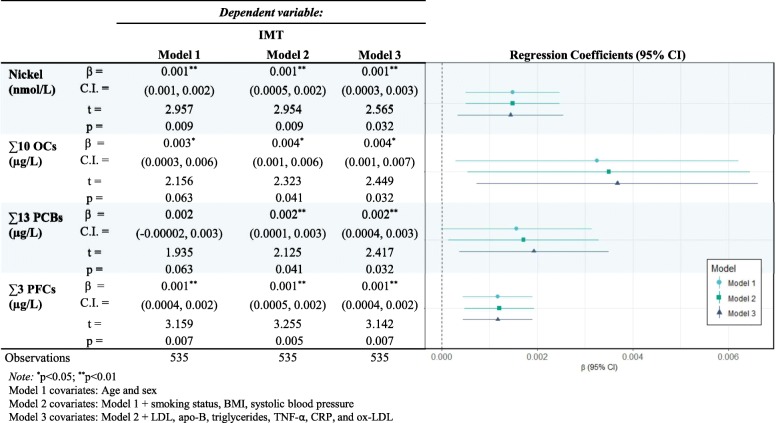
Multivariable linear regression models for the association between Carotid IMT and sum (Σ) of contaminant among participants from the *Nituuchischaayihtitaau Aschii* – Multi-Community Environment-and-Health Study

## Discussion

Using the *Nituuchischaayihtitaau Aschii* – Multi-Community Environment-and-Health Study dataset*,* our findings show that nickel, organochlorines and PFCs are correlated with carotid IMT.

Carotid IMT was regressed on PCA, which illustrated that organochlorines and nickel contaminants may be important risk contributors to CVD. Organochlorine containments found mainly on PC-1 increased cIMT by 0.02 mm per 5-unit PC score change. While this increase may not appear to be clinically relevant, it is important to remember two factors. First, the observed increase in cIMT is solely due to the contaminant loadings on PC-1 after adjustment for multiple covariates. Second, flow rate varies to the 4th power of the lumen diameter, and therefore a reduction in lumen diameter from 4 mm to 3.98 mm represents a flow rate change of 256 mL/min to 250.9 mL/min [40]; hence, slight increases in cIMT have large decreases on blood flow.

Principal component 5, represented mostly by nickel, was shown to have more influence on cIMT than PC-1. Since PC-5 represents 3.1% of the variation within the PCA, other contaminants other than nickel were not assessed in isolation. Nickel was significantly associated with cIMT in all three models. In both human and murine exposures, nickel in air has been shown to be associated with adverse cardiovascular events [41]. Similarly, nickel nanoparticles have been shown to progress atherosclerosis [42]. Though, a cross-sectional analysis of the Prospective Investigation of the Vasculature in Uppsala Seniors (PIVUS) study in Sweden by Lind et al. [21] reported that quintile nickel levels were not associated with cIMT.

Exposure to metals such as cadmium, arsenic, and lead have been shown to increase cardiovascular disease relative risk [43], and in some cases cIMT [44–46]. However, these metals were not highly loaded on our PCA and thus their contribution to the observed variation was low. Similarly, others have investigated the association of cIMT with mercury body burden [20, 22, 47], however, our analysis did not implicate this metal as an important contributor to cIMT. This may be due to the relative importance of the contributions from the other contaminants in the PCA or because other studies assessed contaminants in isolation.

Previous research has found that POPs and PFCs to be positively associated with cIMT [23–25]. Over a 10-year longitudinal study of older adults (70-years and older), the Prospective Investigation of the Vasculature in Uppsala Seniors (PIVUS-study) found that multiple-adjusted PFOS and PFOA corresponded to a 0.011 mm and 0.021 mm change in cIMT, respectively [25]. However, using the same data, a separate cross-sectional analysis by the same group, found no significant association between PFCs and cIMT [26]. Among young adults (12–30 years of age), cIMT significantly increased across PFOS quartiles (0.434 mm, 0.446 mm, 0.458 mm, 0.451 mm) [24]. Several circulating POPs have been shown to be associated with cIMT among seniors, but Lind et al. [23] report that the sum of PCBs concentrations was not associated with cIMT β = 0.001, (95% -0.00004, 0.0033). Though, sum of PCBs concentrations were shown to be significantly inversely associated with grey scale median of the carotid artery intima-media complex (a measure of echogenicity). Our study showed significant and positive associations with nickel, ∑10 OCs, and ∑3 PFCs in all three models, and significant positive associations with the ∑13 PCBs after adjusting for covariates beyond age and sex. These associations were stronger for ∑10 OCs, followed by ∑13 PCBs, ∑3 PFCs, and nickel.

This study has several strengths. Most important, this is the first study to assess the association of a complex mixture of organic, metal, and metalloids on cIMT. The use of PCA allowed us to simultaneously assess all contaminants while narrowing our final examination to the individual contribution of each contaminant of interest to the cIMT. Additionally, the *Nituuchischaayihtitaau Aschii* – Multi-Community Environment-and-Health Study is a comprehensive health survey with a diverse set of quantitative measurements, which aided to reduce the possibility of measurement error. However, several key limitations are also acknowledged. First, this is a cross-sectional analysis with a single assessment of the exposures and outcome, and therefore, temporality and causal relationships cannot be confirmed. Second, smoking has been shown to be associated with persistent organic pollutants such as PCBs and OCPs [48]. Though we adjusted for smoking status, there is still the possibility of residual confounding, as pack-years of cigarettes was not available; and lastly, cIMT increases with age, though we found no appreciable difference in the results when we restricted our sample to include only participates 30 years of age or older. However, stronger associations may be present in aging adults who have longer exposure to body burdens of contaminants.

The role organic and metal contaminants play in CVD has been an area of growing research. Carotid IMT has been shown to be associated with CVD [10], however, the mechanisms by which environmental contaminants exacerbate CVD, and the extent of their contribution, has yet to be fully elucidated. It has been suggested that contaminants such as POPs could influence the pathogenesis of CVDs such as atherosclerosis [23]. With respect to metals, it is possible that the generation of free radicals and subsequent inflammation could also exacerbate CVD, as shown in a murine model of nickel nanoparticle exposure [42]. In the case of metals that do not generate free radicals, such as cadmium, it is possible these metals play an indirect role in reactive oxygen and nitrogen species generation [49], although the underlying mechanism remains unclear [45].

Associations of human body burdens of contaminants with cIMT has been performed on individual contaminants or groups of similar contaminants (e.g., ∑PFCs and ∑PCBs), but this examination has not been performed on complex mixtures of organic and metal/metalloids simultaneously. We used PCA to first determine which components are highly correlated with cIMT, followed by a secondary analysis assessing the individual or groups of components on those identified PCA axes. The work herein is especially important for the Cree from the *Eeyou Istchee* territory, who may have higher body burdens of contaminants than non-Indigenous Canadians. Furthermore, as cIMT was found to be the highest in remote Indigenous Australians when compared to urban Indigenous and non-Indigenous Australians [50], and since Indigenous Canadians have greater health disparities that non-Indigenous Canadians [1, 4], assessing this marker of CVD risk combined with potential environmental contributions is especially important for this region.

## Conclusion

The assessment of complex environmental mixtures and their association to biological outcomes is complicated given the varying possible interactions between the many contaminants that may be present. We have shown that a variety of environmental contaminants are associated with a sub-clinical cardiovascular disease marker carotid IMT, which could possibly contribute to the observed health disparity that exists in Indigenous Canadians. This article also presents a significant step towards assessing complex mixtures and sub-clinical biomarkers, as well as identifying specific contaminants that may ultimately play a role in CVD.

## Data Availability

The data that support the findings of this study are available from the Cree Board of Health and Social Services of James Bay. However, some restrictions apply to the availability of these data, which were used under license for the current study. Data are available from the authors upon reasonable request and with permission of the Cree Board of Health and Social Services of James Bay (http://www.creehealth.org/) and principle investigators.

## Abbreviations

apo-B: Apolipoprotein-B
BMI: Body mass index
cIMT: Carotid intima-media thickness
CVD: Cardiovascular disease
HDL: High-density lipoproteins
hs-CRP: High sensitivity c-reactive protein
ICP-MS: Inductively coupled plasma mass spectrometer
INSPQ: Institut national de Santé Publique du Québec
LDL,: Low-density lipoproteins
OCs: Organochlorines
ox-LDL: Oxidized low-density lipoprotein
PCA: Principal component analysis
PCBs: Polychlorinated biphenyls
PFCs: Perfluorinated compounds
PFHxS: Perfluorohexane sulfonic acid
PFOA: Perfluorooctanoic acid
PFOS: Perfluorooctane sulfonic acid
POPs: Persistent organic pollutants
TNF-α: Tumor necrosis factor

## References

[1] Benjamin EJ, Blaha MJ, Chiuve SE, Cushman M, Das SR, Deo R, et al. Heart disease and stroke statistics—2017 update: a report from the American Heart Association. Circulation. 2017;135. 10.1161/CIR.0000000000000485. 10.1161/CIR.0000000000000485 PMC5408160 28122885

[2] Adelson N (2005). The embodiment of inequity: health disparities in aboriginal Canada. Can J Public Heal.

[3] Reading J (2015). Confronting the growing crisis of cardiovascular disease and heart health among aboriginal peoples in Canada. Can J Cardiol.

[4] Richmond CAM, Cook C (2016). Creating conditions for Canadian aboriginal health equity: the promise of healthy public policy. Public Health Rev.

[5] Anderson I, Robson B, Connolly M, Al-Yaman F, Bjertness E, King A (2016). Indigenous and tribal peoples’ health (the lancet–Lowitja Institute global collaboration): a population study. Lancet.

[6] Gracey M, King M (2009). Indigenous health part 1: determinants and disease patterns. Lancet.

[7] King M, Smith A, Gracey M (2009). Indigenous health part 2: the underlying causes of the health gap. Lancet.

[8] Liberda EN, Tsuji LJS, Martin ID, Cote S, Ayotte P, Dewailly E (2014). Plasma concentrations of persistent organic pollutants in the Cree of northern Quebec.

[9] Nieboer E, Martin ID, Liberda EN, Dewailly E, Robinson E, Tsuji LJS (2017). Body burdens, sources and interrelations of selected toxic and essential elements among the nine Cree first nations of: Eeyou Istchee, James Bay region of northern Quebec, Canada. Environ Sci Process Impacts.

[10] Chambless LE, Heiss G, Folsom AR, Rosamond W, Szklo M, Sharrett AR (1997). Association of coronary heart disease incidence with carotid arterial wall thickness and major risk factors: the atherosclerosis risk in communities (ARIC) study, 1987-1993. Am J Epidemiol.

[11] Lorenz MW, Markus HS, Bots ML, Rosvall M, Sitzer M (2007). Prediction of clinical cardiovascular events with carotid intima-media thickness: a systematic review and meta-analysis. Circulation.

[12] O’Leary DH, Polak JF, Kronmal RA, Manolio TA, Burke GL, Wolfson SK (1999). Carotid-artery intima and media thickness as a risk factor for myocardial infarction and stroke in older adults. N Engl J Med.

[13] Wolf PA, Agostino RBD, Ph D (2011). Carotid intima–media thickness and cardiovascular events. N Engl J Med.

[14] Aguilera I, Dratva J, Caviezel S, Burdet L, De Groot E, Ducret-Stich RE (2016). Particulate matter and subclinical atherosclerosis: associations between different particle sizes and sources with carotid intima-media thickness in the SAPALDIA study. Environ Health Perspect.

[15] Bauer M, Moebus S, Möhlenkamp S, Dragano N, Nonnemacher M, Fuchsluger M (2010). Urban particulate matter air pollution is associated with subclinical atherosclerosis. J Am Coll Cardiol.

[16] Bots ML, Hoes AW, Koudstaal PJ, Hofman A, Grobbee DE (1997). Common carotid intima-media thickness and risk of stroke and myocardial infarction: the Rotterdam study. Circulation.

[17] Kaufman JD, Adar SD, Barr RG, Budoff M, Burke GL, Curl CL (2016). Association between air pollution and coronary artery calcification within six metropolitan areas in the USA (the multi-ethnic study of atherosclerosis and air pollution): a longitudinal cohort study. Lancet (London, England).

[18] Wilker EH, Mittleman MA, Coull BA, Gryparis A, Bots ML, Schwartz J (2013). Long-term exposure to black carbon and carotid intima-media thickness: the normative aging study. Environ Health Perspect.

[19] Ari E, Kaya Y, Demir H, Asicioglu E, Keskin S (2011). The correlation of serum trace elements and heavy metals with carotid artery atherosclerosis in maintenance hemodialysis patients. Biol Trace Elem Res.

[20] Choi AL, Weihe P, Budtz-Jørgensen E, Jørgensen PJ, Salonen JT, Tuomainen T-PP (2009). Methylmercury exposure and adverse cardiovascular effects in Faroese whaling men. Environ Health Perspect.

[21] Lind M, Olsén L, Lind L (2012). Circulating levels of metals are related to carotid atherosclerosis in elderly. Sci Total Environ.

[22] Skoczyńska A, Porȩba R, Steinmentz-Beck A, Martynowicz H, Affelska-Jercha A, Turczyn B (2009). The dependence between urinary mercury concentration and carotid arterial intima-media thickness in workers occupationally exposed to mercury vapour. Int J Occup Med Environ Health.

[23] Lind M, van Bavel B, Salihovic S, Lind L (2012). Circulating levels of persistent organic pollutants (POPs) and carotid atherosclerosis in the elderly. Environ Health Perspect.

[24] Lin CY, Lin LY, Wen TW, Lien GW, Chien KL, Hsu SHJ (2013). Association between levels of serum perfluorooctane sulfate and carotid artery intima-media thickness in adolescents and young adults. Int J Cardiol.

[25] Lind M, Salihovic S, Stubleski J, Kärrman A, Lind L (2018). Changes in plasma levels of perfluoroalkyl substances (PFASs ) are related to increase in carotid intima-media thickness over 10 years – a longitudinal study. Environ Health.

[26] Lind PM, Salihovic S, van Bavel B, Lind L (2017). Circulating levels of perfluoroalkyl substances (PFASs) and carotid artery atherosclerosis. Environ Res.

[27] Lind PM, Lind L (2011). Circulating levels of bisphenol a and phthalates are related to carotid atherosclerosis in the elderly. Atherosclerosis.

[28] Lind L, Lind PM (2012). Can persistent organic pollutants and plastic-associated chemicals cause cardiovascular disease?. J Intern Med.

[29] R Core Team. 2018. R: A Language and Environment for Statistical Computing. http://www.R-project.org/, Vienna, Austria (v3.5.0).

[30] Brownrigg R. 2018. R Package ‘mapdata.’ mapdata Extra Map Databases v2.3.0.

[31] Decorps JP. 2012. R package 'GADMTools.' GADMTools v3.7.2.

[32] Nieboer E, Dewailly E, Johnson-Down L, Sampasa-Kanyinga H, Château-Degat M-L, Egeland GM, Atikessé L, Robinson E TJ, Nieboer E, Dewailly E, Johnson-Down L, Sampasa-Kanyinga H, Château-Degat M-L, et al. 2013. Nituuchischaayihtitaau Aschii Multi-community Environment-and-Health Study in Eeyou Istchee 2005–2009: Final Technical Report. Public Heal Rep Ser 4 Heal Popul Chisasibi QC Cree Board Heal Soc Serv James Ba 1–191. Available: http://www.creehealth.org/sites/default/files/E-and-H Technical Report.pdf [].

[33] Dalla Pozza R, Ehringer-Schetitska D, Fritsch P, Jokinen E, Petropoulos A, Oberhoffer R (2015). Intima media thickness measurement in children: a statement from the Association for European Paediatric Cardiology (AEPC) working group on cardiovascular prevention endorsed by the Association for European Paediatric Cardiology. Atherosclerosis.

[34] Stein James H., Korcarz Claudia E., Hurst R. Todd, Lonn Eva, Kendall Christopher B., Mohler Emile R., Najjar Samer S., Rembold Christopher M., Post Wendy S. (2008). Use of Carotid Ultrasound to Identify Subclinical Vascular Disease and Evaluate Cardiovascular Disease Risk: A Consensus Statement from the American Society of Echocardiography Carotid Intima-Media Thickness Task Force Endorsed by the Society for Vascular Medicine. Journal of the American Society of Echocardiography.

[35] Nieboer E, Robinson E. 2007. Nituuchischaayihtitaau Aschii multi -community environment -and -health longitudinal study in Eeyou Istchee : Eastmain and Wemindji.; doi:978-2-550-61860-7. Available: http://www.creehealth.org/library/online/research/nituuchischaayihtitaau-aschii-multi-community-environment-and-health-0.

[36] Gauch HG. 1982. Multivariate analysis in community ecology.

[37] Green RH. 1979. Sampling design and statistical methods for environmental biologists. Wiley.

[38] Wainman Bruce C., Kesner James S., Martin Ian D., Meadows Juliana W., Krieg Edward F., Nieboer Evert, Tsuji Leonard J. (2016). Menstrual cycle perturbation by organohalogens and elements in the Cree of James Bay, Canada. Chemosphere.

[39] Aickin M, Gensler H (1996). Adjusting for multiple testing when reporting research results: the Bonferroni vs holm methods. Am J Public Health.

[40] Crowley L V. 2011. Essentials of human disease. Jones and Bartlett Publishers.

[41] Lippmann Morton, Ito Kazuhiko, Hwang Jing-Shiang, Maciejczyk Polina, Chen Lung-Chi (2006). Cardiovascular Effects of Nickel in Ambient Air. Environmental Health Perspectives.

[42] Kang GS, Gillespie PA, Gunnison A, Moreira AL, Tchou-Wong K-M, Chen L-C (2011). Long-term inhalation exposure to nickel nanoparticles exacerbated atherosclerosis in a susceptible mouse model. Environ Health Perspect.

[43] Chowdhury R, Ramond A, O’Keeffe LM, Shahzad S, Kunutsor SK, Muka T, et al. 2018. Environmental toxic metal contaminants and risk of cardiovascular disease: systematic review and meta-analysis. BMJ 362:k3310; doi:10.1136/BMJ.K3310. 10.1136/bmj.k3310 PMC6113772 30158148

[44] Chen Y, Hakim ME, Parvez F, Islam T, Rahman AM, Ahsan H (2006). Arsenic exposure from drinking-water and carotid atery intima-medial thickness in healthy young adults in Bangladesh. J Heal Popul Nutr.

[45] Messner B, Knoflach M, Seubert A, Ritsch A, Pfaller K, Henderson B (2009). Cadmium is a novel and independent risk factor for early atherosclerosis mechanisms and in vivo relevance. Arterioscler Thromb Vasc Biol.

[46] Wu Fen, Molinaro Peter, Chen Yu (2014). Arsenic Exposure and Subclinical Endpoints of Cardiovascular Disease. Current Environmental Health Reports.

[47] Salonen JT, Seppänen K, Lakka TA, Salonen R, Kaplan GA (2000). Mercury accumulation and accelerated progression of carotid atherosclerosis: a population-based prospective 4-year follow-up study in men in eastern Finland. Atherosclerosis.

[48] Moon HJ, Lim J, Jee SH (2017). Association between serum concentrations of persistent organic pollutants and smoking in Koreans: a cross-sectional study. J Epidemiol.

[49] Waisberg Michael, Joseph Pius, Hale Beverley, Beyersmann Detmar (2003). Molecular and cellular mechanisms of cadmium carcinogenesis. Toxicology.

[50] Maple-Brown L, Cunningham J, Celermajer DS, O’Dea K (2007). Increased carotid intima-media thickness in remote and urban indigenous Australians: impact of diabetes and components of the metabolic syndrome. Clin Endocrinol.

